# Peptidoglycan ld-Carboxypeptidase Pgp2 Influences *Campylobacter jejuni* Helical Cell Shape and Pathogenic Properties and Provides the Substrate for the dl-Carboxypeptidase Pgp1*[Fn FN2]

**DOI:** 10.1074/jbc.M113.491829

**Published:** 2014-01-06

**Authors:** Emilisa Frirdich, Jenny Vermeulen, Jacob Biboy, Fraser Soares, Michael E. Taveirne, Jeremiah G. Johnson, Victor J. DiRita, Stephen E. Girardin, Waldemar Vollmer, Erin C. Gaynor

**Affiliations:** From the ‡ Department of Microbiology and Immunology, University of British Columbia, Vancouver, British Columbia V6T 1Z3, Canada,; the § Centre for Bacterial Cell Biology, Institute for Cell and Molecular Biosciences, Newcastle University, Newcastle upon Tyne NE2 4AX, United Kingdom,; the ¶ Department of Laboratory Medicine and Pathobiology, University of Toronto, Toronto, Ontario M5S 1A8, Canada, and; the ‖ Department of Microbiology and Immunology, University of Michigan Medical School, Ann Arbor, Michigan 48109

**Keywords:** Campylobacter, Carboxypeptidase, Host-Pathogen Interactions, Microbial Pathogenesis, Peptidoglycan, Bacterial Cell Shape

## Abstract

Despite the importance of *Campylobacter jejuni* as a pathogen, little is known about the fundamental aspects of its peptidoglycan (PG) structure and factors modulating its helical morphology. A PG dl-carboxypeptidase Pgp1 essential for maintenance of *C. jejuni* helical shape was recently identified. Bioinformatic analysis revealed the CJJ81176_0915 gene product as co-occurring with Pgp1 in several organisms. Deletion of *cjj81176_0915* (renamed *pgp2*) resulted in straight morphology, representing the second *C. jejuni* gene affecting cell shape. The PG structure of a Δ*pgp2* mutant showed an increase in tetrapeptide-containing muropeptides and a complete absence of tripeptides, consistent with ld-carboxypeptidase activity, which was confirmed biochemically. PG analysis of a Δ*pgp1*Δ*pgp2* double mutant demonstrated that Pgp2 activity is required to generate the tripeptide substrate for Pgp1. Loss of *pgp2* affected several pathogenic properties; the deletion strain was defective for motility in semisolid agar, biofilm formation, and fluorescence on calcofluor white. Δ*pgp2* PG also caused decreased stimulation of the human nucleotide-binding oligomerization domain 1 (Nod1) proinflammatory mediator in comparison with wild type, as expected from the reduction in muropeptide tripeptides (the primary Nod1 agonist) in the mutant; however, these changes did not alter the ability of the Δ*pgp2* mutant strain to survive within human epithelial cells or to elicit secretion of IL-8 from epithelial cells after infection. The *pgp2* mutant also showed significantly reduced fitness in a chick colonization model. Collectively, these analyses enhance our understanding of *C. jejuni* PG maturation and help to clarify how PG structure and cell shape impact pathogenic attributes.

## Teaser

**Background:** *C. jejuni* helical shape is important to pathogenesis.

**Results:** Deletion of *pgp2* results in loss of *C. jejuni* helical shape and change in peptidoglycan structure and pathogenic properties.

**Conclusion:** Pgp2 is a ld-carboxypeptidase cleaving peptidoglycan tetrapeptides to tripeptides.

**Significance:** Characterization of enzymes involved in *C. jejuni* peptidoglycan and cell shape maintenance is crucial to the understanding of fundamental properties of this organism.

## Introduction

The Gram-negative bacterium *Campylobacter jejuni* is a helical organism exhibiting characteristic corkscrew motility. Despite fastidious growth requirements, it is a prevalent zoonotic organism existing in the intestinal tract of birds and other animal species (1–3) and is a leading cause of human bacterial diarrheal disease worldwide (4, 5). The pathogens *C. jejuni* and *Helicobacter pylori* are the most well studied members of the ϵ-Proteobacteria, a relatively poorly characterized class of a wide variety of bacteria, including numerous extremophiles. Most members of the ϵ-Proteobacteria examined to date display helical or curved morphology (6).

It has been hypothesized that the helical morphology of *C. jejuni* and its polar flagella are responsible for its enhanced ability in comparison with rod-shaped bacteria to move through viscous substances, such as the mucus layer of the gastrointestinal tract (7). In most bacteria, morphology is maintained by the peptidoglycan (PG)^4^ sacculus (8–10). This is also the case for *C. jejuni* because deletion of the *C. jejuni* PG dl-carboxypeptidase Pgp1 (peptidoglycan peptidase 1) resulted in loss of helical shape and alterations in a number of stress survival and host-related attributes (11). Pgp1, which cleaves monomeric PG tripeptides to dipeptides, was identified in a calcofluor white (CFW)-based screen for mutants with altered cell envelope and pathogenic properties (11). CFW is a compound reacting with β1–3 and β1–4 carbohydrate linkages and fluoresces under long wave UV light (12, 13). The *C. jejuni* carbohydrate involved in CFW hypo- *versus* hyper-reactivity has not been clarified, although it has been shown not to directly correlate with the characterized *C. jejuni* cell surface polysaccharides: the capsular polysaccharide, lipooligosaccharide, *O*-linked flagellar glycoproteins, or *N*-linked glycoproteins (14). CFW does bind isolated PG,^5^ so it is possible that it is the accessibility of CFW to binding sites on periplasmic PG molecules that determines CFW reactivity differences. Despite having changes in PG composition and cell morphology, the rod-shaped Δ*pgp1* mutant showed no apparent differences in comparison with wild type in other cell surface structures, growth characteristics, stress survival, and antimicrobial compound sensitivity. The rod-shaped Δ*pgp1* was altered in all aspects of the *C. jejuni* life cycle: transmission (with reduced motility in semisolid agar and decreased biofilm formation), colonization (exhibiting a 3 log-fold reduction in chick colonization), and host cell interactions (increased activation of the cytoplasmic human nucleotide-binding oligomerization domain 1 (Nod1) receptor by Δ*pgp1* PG and increased secretion of the IL-8 chemokine in epithelial cell infections). Surprisingly, the Δ*pgp1* mutant was not defective for host cell attachment or invasion *in vitro* even in the presence of media of higher viscosity, although this may not hold true *in vivo* in intestinal mucus.

We are just beginning to understand fundamental aspects of PG structure and biosynthesis in this important human pathogen, along with the unique factors involved in sculpting its helical morphology. The PG of *C. jejuni* and all Gram-negative organisms is composed of glycan strands of alternating precursors of β1–4-linked *N*-acetylglucosamine (GlcNAc) and *N*-acetylmuramic acid (MurNAc) residues, which are connected by short peptides. The peptides are synthesized as pentapeptides (l-Ala-d-isoglutamic acid-*meso*-DAP-d-Ala-d-Ala), which can be cross-linked to dimers or oligomers by dd-transpeptidases or trimmed to tetra-, tri-, or dipeptides by dd-, ld-, and dl-carboxypeptidases, respectively. The mature PG is composed of a mixture of subunits called muropeptides that differ in the number and length of connected peptides. The muropeptide profile of *C. jejuni* indicates very low levels of pentapeptides and a high abundance of trimmed stem peptide species (11).

Bioinformatic analyses identified three putative penicillin-binding proteins (PBPs) or PG synthases in *C. jejuni*: CJJ81176_0536 with similarity to the bifunctional synthase PBP1A encoding PG glycosyltransferase and dd-transpeptidase domains and CJJ81176_0680 and CJJ81176_0550 with homology to the monofunctional transpeptidases PBP2 (involved in cell elongation) and PBP3 (involved in cell division), respectively. PG biosynthesis also requires the activity of hydrolases, allowing for the insertion of newly attached material in cell elongation and the separation of cells in cell division (8, 9, 15, 16). *C. jejuni* does have homologs of the lytic transglycosylases Slt (CJJ81176_0859) and MltD (CJJ81176_0673) as well as the amidase AmiA (CJJ81176_1285) that probably also play a role in these processes. PG hydrolases with endo- and carboxypeptidase activity are also important in sculpting the PG layer to determine cell shape (9). *C. jejuni* does not have predicted homologs of the low molecular weight PBPs with dd-endo- or carboxypeptidase activity but does have homologs of the *H. pylori* Csd1 endopeptidase and of the Csd3/HdpA endo-/carboxypeptidase that act on pentapeptide-containing muropeptides (17, 18). In *H. pylori*, deletion of *csd1* and *csd3* results in curved rod morphologies with enhanced levels of PG cross-linking (17).

This study describes the identification and characterization of the *C. jejuni* ld-carboxypeptidase Pgp2 (peptidoglycan peptidase 2). Pgp2 trims tetrapeptides to tripeptides and creates the substrate for Pgp1, with both enzyme activities required for *C. jejuni* helical morphology. The importance of Pgp2 in a number of properties related to pathogenesis is also described, most notably its role in motility, the capacity to form biofilms, the manner in which *C. jejuni* is recognized by human cells, and its ability to colonize a zoonotic avian host.

## EXPERIMENTAL PROCEDURES

### 

#### 

##### Bacterial Strains and Growth Conditions

Bacterial strains and plasmids used in this study and their construction are described in the supplemental Experimental Procedures. Unless otherwise stated, *C. jejuni* strains were grown at 38 °C in Mueller-Hinton (MH; Oxoid) broth or 8.5% (w/v) agar supplemented with vancomycin (10 μg/ml) and trimethoprim (5 μg/ml) (unless otherwise indicated) under microaerobic/capnophilic conditions (6% O_2_, 12% CO_2_) in a Sanyo trigas incubator for plates or using the Oxoid CampyGen system for broth cultures. Growth media were supplemented with chloramphenicol (20 μg/ml) or kanamycin (Km; 50 μg/ml) where appropriate. *Escherichia coli* strains used for plasmid construction were grown at 37 °C in Luria-Bertani (LB; Sigma) broth or 7.5% agar (w/v) and supplemented with ampicillin (100 μg/ml), chloramphenicol (15 μg/ml), or Km (25 μg/ml), as necessary.

##### Microscopy

Transmission electron microscopy (TEM) was carried out on samples fixed in a final concentration of 2.5% (v/v) glutaraldehyde from overnight broth cultures, as described previously (11). Samples were visualized on a Hitachi H7600 TEM equipped with a side mount AMT Advantage (1-megapixel) CCD camera (Hamamatsu ORCA) at the UBC Bioimaging facility (University of British Columbia, Vancouver, Canada).

##### Phenotypic Characterization; Motility, Biofilm Formation, and CFW Assays

Phenotypic assays were carried out with strains that had been grown in shaking MH-trimethoprim-vancomycin broth for 18 h. Motility, biofilm formation, and CFW fluorescence were assayed as described previously (11).

##### Peptidoglycan Isolation and Muropeptide Analysis

*C. jejuni* strains were passaged once from frozen stocks and then passaged to 20–25 MH plates and grown for 20 h to obtain log phase bacteria at a final OD of 200–600. Cells were collected into cold MH broth by scraping, harvested by centrifugation at 8,000 × *g* for 15 min, and then resuspended in 6 ml of ice-cold H_2_O. Cells were lysed by dropwise addition to 6 ml of 8% SDS boiling under reflux. PG was purified from the cell lysate as described (19). For muropeptide analysis, the isolated PG was digested with the muramidase cellosyl (kindly provided by Hoechst, Frankfurt, Germany), and the resulting muropeptides were reduced with sodium borohydride and separated by HPLC, as described (19). Muropeptide structures were assigned (i) based on comparison with retention times of known muropeptides from *C. jejuni* (11) and (ii) by mass spectrometry (MS). For MS analysis, muropeptide fractions were collected, concentrated in a SpeedVac, acidified by 1% trifluoroacetic acid, and analyzed by offline electrospray mass spectrometry on a Finnigan LTQ-FT mass spectrometer (ThermoElectron, Bremen, Germany) at the Newcastle University Pinnacle facility as described (20).

##### Expression, Purification, and Enzymatic Activity of Pgp2

The *C. jejuni* 81-176 *pgp2* gene was cloned for expression in *E. coli* without its signal peptide (amino acids 19–325 of the protein) and stop codon, in frame with the C-terminal His_6_ tag of the pET28a vector, generating plasmid pEF70. A detailed description of the cloning of the expression construct, expression, and purification protocol is included in the supplemental Experimental Procedures. For enzyme assays, the purified protein was dialyzed against 0.05 m Tris-Cl, pH 7.5, containing 0.3 m NaCl and 20% glycerol. Purified Δ*pgp2* PG (1 mg/ml) was incubated with Pgp2-His_6_ (5 μm) in 0.02 m NaH_2_PO_4_, pH 4.8, 0.1 m NaCl for 4 h at 37 °C on a Thermomixer at 750 rpm. A control sample received no enzyme. The samples were incubated with 10 μg of cellosyl (Hoechst, Frankfurt am Main, Germany) for 18 h, boiled for 10 min, and centrifuged at room temperature for 15 min at 16,000 × *g*. The muropeptides present in the supernatant were reduced with sodium borohydride and analyzed by HPLC, as described (19).

##### In Vitro Invasion and Intracellular Survival in Epithelial Cell Lines

The human epithelial cell lines CaCo2 and INT407 were used for *C. jejuni* infections. Medium used for growth of the cell lines was as directed by the ATCC. Cells were seeded into 24-well tissue culture plates at semiconfluence at ∼5 × 10^5^ and ∼1 × 10^5^ cells/ml for CaCo2 and INT407 cells, respectively. Infections were carried out as described (11). To test the levels of invasion in media of higher viscosity to mimic intestinal mucus, carboxymethylcellulose (CMC) (Sigma) was used. Infections were carried out as above using the INT407 cell line. The bacterial inoculum was added in MEM containing 0, 0.6 (141 centipoise (cP)), 1, and 2% CMC. Levels of adhered and invaded bacteria at the 1 and 3 h time points were determined by washing and lysing the cells and plating for cfu/ml as described (11).

##### Epithelial Cell Responses and Nod Activation Assays

Luciferase assays were performed as described (21). Briefly, HEK293T cells were transfected overnight with 75 ng of NF-κB luciferase reporter plasmid (Igκ-luc, Invitrogen) and either human Nod1 (2 ng) or Nod2 (0.3 ng). The empty vector (pcDNA3.1, Invitrogen) was used to balance the transfected DNA concentration. At the same time, either 0.2 or 2 μg of *C. jejuni* 81-176, Δ*pgp2*, or Δ*pgp2c* PG muropeptides were added, and the NFκB-dependent luciferase activation was then measured following 18–24 h of co-incubation. To generate muropeptides, a 2 mg/ml stock of PG of each strain was digested with mutanolysin at 125 units/100 μl overnight at 38 °C. Positive controls were tripeptide l-Ala-γ-d-Glu-*meso*-DAP (5 μg/ml) and muramyldipeptide (MDP) (10 μg/ml) for Nod1 and Nod2 assays, respectively. Data are representative of four independent experiments, each performed in triplicate.

##### Interleukin-8 Quantification

The concentration of IL-8 secreted by INT407 human epithelial cells either left uninfected or infected with *C. jejuni* wild-type strain 81-176, Δ*pgp2*, or Δ*pgp2c* was assayed using the human IL-8 ELISA kit (Invitrogen) as described previously (11).

##### Chick Colonization

Chick colonization was performed as described previously (11, 22, 23), with an infective dose of 10^4^ cfu. Briefly, 1-day-old chicks were colonized with 1 × 10^4^ cfu via oral gavage. Seven days postcolonization, cecal contents were removed, diluted in PBS, and plated on *Campylobacter*-selective media (MH agar supplemented with vancomycin (40 μg/ml), trimethoprim (10 μg/ml), cefoparazone (40 μg/ml), and cyclohexamide (100 μg/ml)) and allowed to grow at 42 °C under microaerobic conditions until countable colonies appeared (∼2–3 days). cfu counts were standardized to g of cecal content. Chicken experiments were carried out under protocol 10462 approved by the University of Michigan Committee on Care and Use of Animals.

## RESULTS

### 

#### 

##### The pgp2 Gene Was Identified as Interacting with pgp1 by STRING Analysis and Deleted by In-frame Deletion by Selection on d-Ala

To discover additional *C. jejuni* proteins involved in maintaining helical shape, putative protein interaction partners for Pgp1 were identified bioinformatically by STRING (Search Tool for the Retrieval of Interacting Genes/Proteins) analysis. The *pgp1* (*cjj81176*_*1344*) gene product was found to be associated with high confidence to *cjj81176*_*0915* by their co-occurrence in numerous organisms. In addition, the two genes were found to be in the immediate neighborhood of each other (within 300 bp) on the genome in the ϵ-Proteobacteria *Nitratiruptor* sp. SB155 and *Nautilia profundicola*, the δ-Proteobacterium *Desulfococcus deovorans,* and a member of the Aquificae *Persephonella marina*. The *0915* gene was named *pgp2* (peptidoglycan peptidase 2) to describe its function and identification as the second *C. jejuni* PG peptidase to be characterized.

**FIGURE 1.**
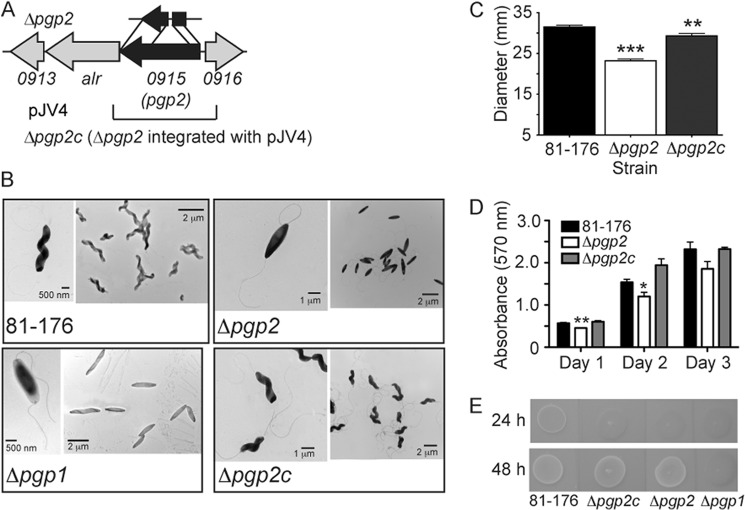
***C. jejuni* 81-176 *pgp2* gene locus, *pgp2* mutant straight morphology, and defects in motility, biofilms, and CFW reactivity.** *A*, the *pgp2* mutant was constructed by deleting a 439-bp internal fragment of *pgp2*; the approximate location of this deletion is shown *above* the gene cluster and is denoted by the Δ*pgp2* strain designation. The region cloned into the integrative vector pRRC (pJV4; Cm^R^) used for complementation is shown *below* the gene cluster. *B*, negatively stained TEM images of the helical *C. jejuni* 81-176 strain, the straight Δ*pgp1* (described previously (11)) and Δ*pgp2* mutant strains with intact flagella, and complemented strain Δ*pgp2*c with restored helical morphology. *C*, Δ*pgp2* exhibited a 26.3% decrease in motility, as assayed by measuring halo diameters in soft agar plates. S.E. (*error bars*) was calculated from 10 measurements. *D*, Δ*pgp2* was defective for biofilm formation, which was complemented in Δ*pgp2*c. Biofilm formation was assessed by crystal violet staining of standing cultures in borosilicate tubes and quantification of dissolved crystal violet at 570 nm. S.E. values were calculated from triplicate cultures and are representative of three independent experiments. *E*, Δ*pgp2* was hypofluorescent relative to wild-type 81-176 after 24 h but not 48 h of growth on plates containing 0.002% CFW (in contrast, Δ*pgp1* remained hypofluorescent after 48 h of growth (11)). Δ*pgp2* hypofluorescence was not restored by complementation. *, statistically significant difference using the unpaired Student's *t* test, with *, **, and *** indicating *p* < 0.05, *p* < 0.01, and *p* < 0.0001, respectively.

Pgp2 is a 325-amino acid protein with a molecular mass of 37.8 kDa that was annotated as a hypothetical protein. It has a predicted N-terminal signal sequence by SIGNALP that is cleaved between amino acids 18 and 19. Conserved domain searches and the threading program PHYRE identified a ld-transpeptidase-like catalytic domain (YkuD; pfam0734) spanning amino acids 67–196. In other organisms, ld-transpeptidases have been shown to give rise to an alternative type of PG cross-linking between the third amino acid (*meso*-DAP) residues of the stem peptides and are involved in attaching the outer membrane lipoprotein Lpp to the PG sacculus. However, neither *meso*-DAP-*meso*-DAP cross-links nor bound lipoproteins have been identified in *C. jejuni* (11), suggesting that Pgp2 might catalyze a different reaction. Unlike *pgp1*, the *pgp2* gene product is not restricted to helical and vibrioid bacteria and is conserved in both Gram-negative and Gram-positive organisms. The *H. pylori* homolog is described by Sycuro *et al.* (24). A putative alanine racemase gene (*alr*) is encoded directly downstream of *pgp2* in *C. jejuni* strain 81-176 (Fig. 1*A*). Alr catalyzes the conversion of l- to d-Ala, an essential component of the PG stem peptide.

To study the role of *pgp2* in *C. jejuni* PG biosynthesis and morphology, we initially constructed a deletion mutant in strain 81-176 by replacing a portion of the gene with a non-polar *aphA3* Km resistance cassette. This produced a strain with completely straight morphology but also modest growth defects (data not shown). The characteristic *C. jejuni* helical cell shape, but not the growth defects, could be complemented by insertion of the pRRC-*pgp2* (pJV4) complementing plasmid construct (Fig. 1*A*). This suggested minor polar activity on *alr* (predicted to be essential), which was confirmed by full complementation of this initial *pgp2* mutant with a pRRC derivative carrying both *pgp2* and the downstream *alr* (not shown). We thus decided to construct an unmarked, in-frame deletion of *pgp2*. An *aphA3(Km)*-sacB cassette adapted *for H. pylori* (pKSFII) (25) was first used to create a marked deletion of *pgp2*. We have now confirmed *alr* deletion to be lethal but also fully rescued by supplementation of growth medium with d-Ala.^6^ The *aphA3(Km)*-sacB-disrupted *pgp2* mutant strain only grew on plates containing d-Ala, indicating full polar effects on *alr*. Although sucrose counterselection proved unsuccessful, we reasoned that growth without d-Ala could be used instead as a counterselection for the creation of an unmarked in-frame deletion. Transformants with the *aphA3-sacB* cassette replaced by an in-frame deletion of *pgp2* were selected by growth on media without d-Ala to create strain Δ*pgp2* (Fig. 1*A*). The complemented strain created by integration of pRRC carrying *pgp2* (pJV4) into the rRNA spacer region of Δ*pgp2* is designated Δ*pgp2c* (Fig. 1*A*). A Δ*pgp1*Δ*pgp2* double mutant was also generated to examine the loss of both genes upon *C. jejuni* PG biosynthesis.

##### Loss of pgp2 Affects Helical Morphology and Has Effects on Motility, Biofilm Formation, and CFW Reactivity

The Δ*pgp2* mutant displayed a straight morphology and loss of the characteristic *C. jejuni* helical cell shape (Fig. 1*B*). This was similar to Δ*pgp1*; however, Δ*pgp2* appeared to have more of an elliptical shape than Δ*pgp1*, with a larger cell width at midcell and more tapered ends than the more rectangle-shaped Δ*pgp1*. Complementation restored the wild-type helical shape (Fig. 1*B*).

The motility of Δ*pgp2* in soft agar plates measured by halo diameter was, on average, 73.7% of wild type (7.8% lower than the average motility of Δ*pgp1*) (11) (Fig. 1*C*). No flagellar structural defects were observed for Δ*pgp2* (Fig. 1*B*). Complementation restored motility to 93.0% of wild type (Fig. 1*C*). Crystal violet assays were used to assess biofilm formation over 3 days. Biofilm levels of Δ*pgp2* were ∼1.2-, 1.3-, and 1.2-fold lower in comparison with wild type at days 1, 2, and 3, respectively (Fig. 1*D*). Biofilm production was restored in Δ*pgp2*c (Fig. 1*D*). Changes in CFW reactivity typically correlate with biofilm formation differences (11, 14, 26); therefore, CFW reactivity of Δ*pgp2* was tested. The Δ*pgp2* mutant was hyporeactive relative to wild type after 24 h of growth on CFW, but after 48 h, it showed wild-type fluorescence levels (Fig. 1*E*). This was in contrast to Δ*pgp1*, which maintains hypo-fluorescence for 48 h. Wild-type CFW reactivity of Δ*pgp2* was not restored by complementation.

**FIGURE 2.**
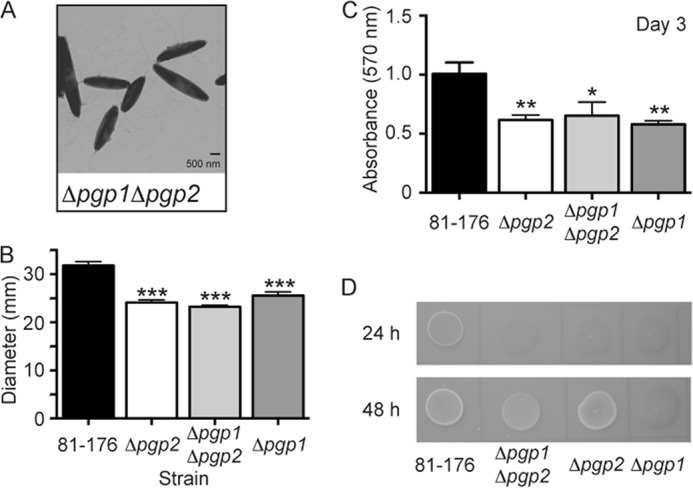
***C. jejuni* 81-176 Δ*pgp1*Δ*pgp2* double mutant straight morphology and defects in motility, biofilms, and CFW reactivity.** *A*, negatively stained TEM images of the straight Δ*pgp1*Δ*pgp2* double mutant strain with intact flagella showing a similar morphology to Δ*pgp2. B*, Δ*pgp1*Δ*pgp2* exhibited a 27.0% decrease in motility identical to that of Δ*pgp2*, as assayed by measuring halo diameters in soft agar plates. S.E. (*error bars*) was calculated from 10 measurements. *C*, Δ*pgp1*Δ*pgp2* was defective for biofilm formation to the same extent as both Δ*pgp1* and Δ*pgp2* and is shown after 3 days. Biofilm formation was assessed by crystal violet staining of standing cultures in borosilicate tubes and quantification of dissolved crystal violet at 570 nm. S.E. values were calculated from triplicate cultures and are representative of three independent experiments. *D*, on plates containing 0.002% CFW, Δ*pgp1*Δ*pgp2* was hypofluorescent relative to wild-type 81-176 after 24 h (like Δ*pgp1* and Δ*pgp2*) but at 48 h of growth showed intermediate fluorescence to Δ*pgp1* and Δ*pgp2*. *, statistically significant difference using the unpaired Student's *t* test, with *, **, and *** indicating *p* < 0.05, *p* < 0.01, and *p* < 0.0001, respectively.

The Δ*pgp2* mutant exhibited no differences in comparison with wild type for growth, stress survival, capsule and lipooligosaccharide migration on acrylamide gels, membrane protein composition, and sensitivity to antimicrobial compounds (supplemental Table S1). The Δ*pgp1*Δ*pgp2* double mutant exhibited an identical morphology, motility, and biofilm phenotype as Δ*pgp2,* suggesting *pgp2* as epistatic to *pgp1*, with its CFW reactivity at 48 h being intermediate between that of Δ*pgp1* and Δ*pgp2* (Fig. 2).

##### The Δpgp2 Peptidoglycan Muropeptide Profile Is Distinct from That of Δpgp1, Displaying an Increase in Tetrapeptides and Complete Lack of Tripeptides, with the Muropeptide Profile of Δpgp1Δpgp2 Identical to That of Δpgp2

**FIGURE 3.**
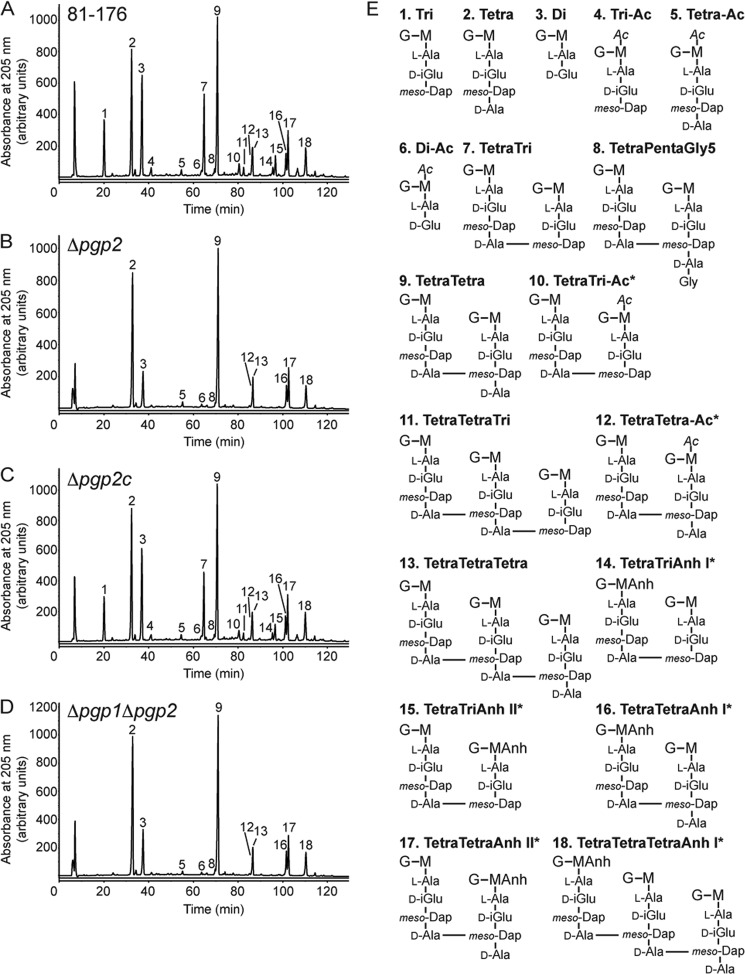
**HPLC elution profile of *C. jejuni* muropeptides and proposed muropeptide structures.** Purified PG was digested with cellosyl, and the resulting muropeptides were reduced with sodium borohydride and separated on a Prontosil 120-3-C18 AQ reverse-phase column. HPLC profiles are shown for *C. jejuni* wild-type 81-176 (*A*), Δ*pgp2* (*B*), the complement Δ*pgp2*c (*C*), and the double mutant Δ*pgp1*Δ*pgp2* (*D*). Peak numbers correspond to the main muropeptide fractions (11) whose structures are shown in *E. G*, *N*-acetylglucosamine; *M*, reduced *N*-acetylmuramic acid; d-*iGlu*, d-isoglutamic acid; *Ac*, O-acetyl groups at the C-6 hydroxyl group of MurNAc; *Anh*, 1,6-anhydro group at MurNAc. *, it is not known on which MurNAc residue the modification occurs.

The bioinformatic association of Pgp2 with the PG dl-carboxypeptidase Pgp1, the presence of putative ld-transpeptidase domains in Pgp2, and the shape phenotype of Δ*pgp2* all indicated that Pgp2 is involved in PG biosynthesis or modification. PG from the wild-type 81-176 strain, Δ*pgp2* mutant, complemented mutant (Δ*pgp2*c), and Δ*pgp1*Δ*pgp2* double mutant were isolated, and the muropeptide profiles were analyzed by HPLC (Fig. 3, *A–D*, Table 1, and supplemental Table S2). The Δ*pgp2* mutant displayed a complete absence of tripeptides and an increase in tetrapeptides, both in the monomeric and cross-linked dimeric and trimeric forms. This suggested that Pgp2 is an ld-carboxypeptidase trimming tetrapeptides to tripeptides and that Pgp2 is the only enzyme with such activity present in *C. jejuni*. The only other significant change in the muropeptide profile of the *pgp2* mutant was a decrease in the dipeptides. The Δ*pgp2c* strain exhibited full complementation to the wild-type profile. The muropeptide profile of the Δ*pgp1*Δ*pgp2* double mutant was virtually identical to that of Δ*pgp2* (Fig. 3, *B* and *D*; see Table 1 for a comparison of the muropeptide composition of Δ*pgp1*Δ*pgp2* with that of Δ*pgp1* and Δ*pgp2*).

**TABLE 1:** **Summary of the muropeptide composition of *C. jejuni* wild-type 81-176, Δ*pgp2*, Δ*pgp2* complement (Δ*pgp2*c), Δ*pgp1*Δ*pgp2* double mutant, and Δ*pgp1* and the resultant Δ*pgp2* PG profiles of Pgp2 activity assays consisting of Δ*pgp2* PG incubated with and without Pgp2 enzyme preparations** Values represent the percentage area of each muropeptide from supplemental Table S2 calculated to give a total of 100%. Values indicated with an asterisk represent a greater than or equal to 20% difference in comparison with *C. jejuni* wild-type 81-176 or Δ*pgp2* PG to which no enzyme was added, and boldface, asterisked values indicate a greater than or equal to 30% change.

Muropeptide species	Percentage of peak area						
	In *C. jejuni* strains					Following incubation with Δ*pgp2* PG	
	81-176	Δ*pgp2*	Δ*pgp2c*	Δ*pgp1*Δ*pgp2*	Δ*pgp1**^a^*	Δ*pgp2* PG + buffer	Δ*pgp2* PG + Pgp2
	%	%	%	%	%	%	%
**Monomers**							
Di	15.3	9.1*	14.8	10.2*	5.9	9.8	11.0
Tri	8.9	0.0*	7.4	0.0*	34.4	0.0	39.0*
Tetra	17.7	31.7*	19.6	30.7*	3.1	30.9	1.4*
Anhydro						0.0	6.2*
Total	41.6	40.8	41.8	40.9	43.4	40.7	51.4*

**Dimers**							
TetraTri	16.0	0.0*	14.2	0.0*	37.0	0.0	24.5*
TetraTetra	31.1	46.8*	33.2	46.9*	14.7	49.1	16.1*
Tetra PentaGly^5^	0.7	0.8	0.7	1.0*	0.7	1.0	1.0
Anhydro	12.5	12.1	12.7	11.8	11.7	12.3	7.0*
Total	47.9	47.6	48.0	47.9	52.5	50.1	41.5

**Trimers**							
TetraTetraTri	1.0	0.0*	0.8	0.0*	1.9	0.0	2.3*
TetraTetraTetra	9.5	11.6	9.4	11.2	2.3	9.2	4.8*
Total	10.5	11.6	10.2	11.2	4.2	9.2	7.1
Dipeptides (Total)	15.3	9.1*	14.8	10.2*	5.9	9.8	11.0
Tripeptides (Total)	17.3	0.0*	14.7	0.0*	53.6	0.0	52.0*
Tetrapeptides (Total)	67.0	90.5*	70.2	89.3*	40.2	89.7	36.5*
Pentapeptides (Total)	0.4	0.4	0.4	0.5	0.4	0.5	0.5
Anhydro chain ends (Total)	7.9	8.0	8.0	7.8	6.4	8.1	10.5*
Average chain length	12.7	12.5	12.5	12.8	15.7	12.4	9.5*
Degree of cross-linkage	31.0	31.6	30.8	31.5	29.0	31.2	25.5
Percentage of peptides in cross-links	58.4	59.2	58.2	59.2	56.6	59.3	48.6

*^a^* Values for Δ*pgp1* are as published previously (11). For characterization of the differences between wild type and Δ*pgp1*, see Frirdich *et al.* (11). Differences between the wild type and Δ*pgp1* are not highlighted with boldface and asterisked values.

##### Pgp2 Is an ld-Carboxypeptidase Cleaving Monomeric and Cross-linked Disaccharide Tetrapeptides to Tripeptides

**FIGURE 4.**
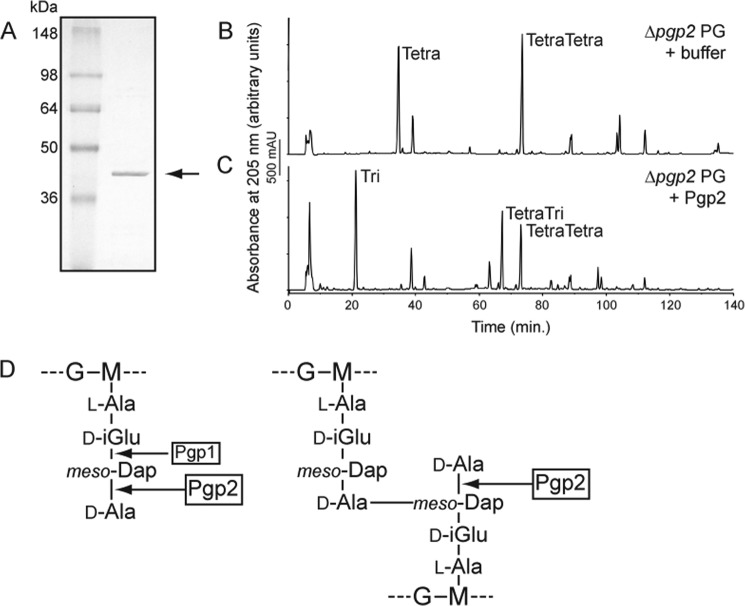
**Pgp2 has ld-carboxypeptidase activity on Δ*pgp2* PG, cleaving monomeric and cross-linked disaccharide tetrapeptides to tripeptides.** *A*, SDS-PAGE analysis of affinity-purified Pgp2 with a predicted molecular mass of 37.0 kDa, indicated by an *arrow*. Shown are HPLC chromatograms of Δ*pgp2* PG (*B*) and Δ*pgp2* PG incubated with purified Pgp2, followed by cellosyl digestion and reduction with sodium borohydride (*C*). Peaks corresponding to monomeric disaccharide tripeptide (*Tri*) and disaccharide tetrapeptide (*Tetra*) and dimeric bis-disaccharide tetratripeptide (*TetraTri*) and bis-saccharide tetratetrapeptide (*TetraTetra*) are indicated. *D*, schematic diagram of the Pgp1 (determined in Ref. 11); Pgp2 carboxypeptidase cleavage sites are indicated with an *arrow*. Note that Pgp2 hydrolyzes tetrapeptides, and Pgp1 hydrolyzes tripeptides. *G*, *N*-acetylglucosamine; *M*, *N*-acetylmuramic acid; d-*iGlu*, d-isoglutamic acid.

To demonstrate biochemically that Pgp2 is an ld-carboxypeptidase trimming tetrapeptides to tripeptides, the Pgp2 protein was expressed in *E. coli* without its putative signal peptide and with a C-terminal His_6_ tag and purified to ∼98% purity (Fig. 4*A*). The purified protein was incubated with PG from the Δ*pgp2* mutant strain and subjected to digestion with cellosyl, and the resulting muropeptides were analyzed by HPLC (Fig. 4, *B* and *C*, Table 1, and supplemental Table S2). Incubation with reaction buffer only caused no change in the muropeptide profile. The addition of Pgp2 resulted in a 94% reduction of monomeric tetrapeptides, a 68% reduction in dimeric tetrapeptides, and a 47% reduction in trimeric tetrapeptides (Table 1). This analysis showed that Pgp2 is an ld-carboxypeptidase trimming tetrapeptides to tripeptides with a higher activity on monomeric peptides than cross-linked peptides (Table 1 and Fig. 4*D*).

##### The Peptidoglycan Changes in Δpgp2 Result in a Dramatic Decrease in Human Nod1 Signaling; the Δpgp2 Mutant Strain Shows No Change in Intracellular Survival or IL-8 Secretion from Epithelial Cells but Is Defective for Chick Colonization in Vivo

**FIGURE 5.**
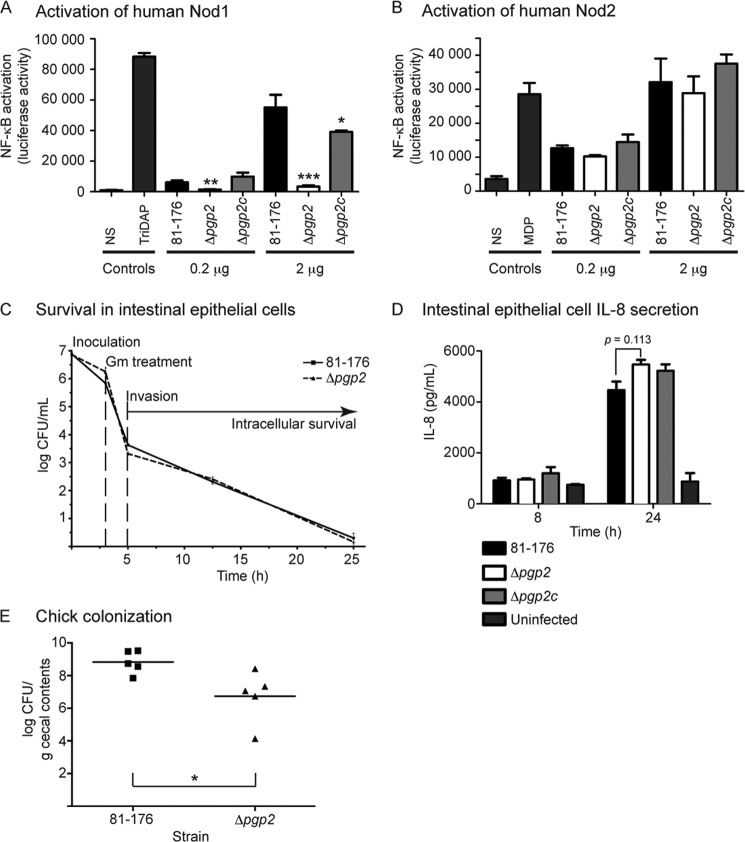
**The effect of *pgp2* deletion on host-related phenotypes.** *A* and *B*, PG isolated from the Δ*pgp2* mutant shows reduced human Nod1 activation and no change in human Nod2 stimulation. To assay the change in the ability of *C. jejuni* Δ*pgp2* PG to activate Nod proteins, human embryonic kidney cells (HEK293T) were co-transfected with either 0.2 or 2 μg of *C. jejuni* 81-176, Δ*pgp2*, or Δ*pgp2c* PG, vectors for a nuclear factor-κB (NF-κB) luciferase reporter, and either Nod1 (*A*) or Nod2 (*B*). Nod activation was determined by measuring the activity of a NF-κB luciferase reporter in comparison with the non-stimulated (*NS*) negative control. Positive controls used were tripeptide l-Ala-γ-d-Glu-*meso*-DAP (*TriDAP*) and MDP. Data represent the mean ± S.E. of four independent experiments. *C*, invasion and intracellular survival ability of the Δ*pgp2* strain in the INT407 epithelial cell line was assessed by a gentamicin (*Gm*) protection assay and showed no change in comparison with wild type. Gentamicin was added 3 h postinfection with the *C. jejuni* wild-type 81-176 and Δ*pgp2* strains. After 2 h, the gentamicin was washed off, and the cells were incubated with fresh MEM containing 3% FBS and a low dose of gentamicin. cfu were determined for each well by lysing the cells with water and plating the dilutions onto MH-trimethoprim-vancomycin plates. Data represent the mean ± S.E. of three independent experiments. *D*, *pgp2* deletion has no effect on IL-8 secretion in the INT407 epithelial cell line. ELISA was used to quantify IL-8 levels secreted by uninfected INT407 epithelial cell lines and cells infected for 8 and 24 h with *C. jejuni* wild-type 81-176, Δ*pgp2,* and Δ*pgp2*c strains. Data represent the mean ± S.E. (*error bars*) of three independent experiments. *E*, the Δ*pgp2* mutant strain is defective for chick colonization. Each *point* represents the log cfu/g cecal contents of an individual chick 7 days postcolonization with 10^4^ cfu of the indicated *C. jejuni* strains. The geometric mean is denoted by a *black bar*. *, statistically significant difference using the unpaired Student's *t* test, with *, **, and *** indicating *p* < 0.05, *p* < 0.01, and *p* < 0.0001, respectively.

Host cell cytoplasmic Nod-like receptors of the innate immune system recognize PG. Human Nod1 preferentially recognizes DAP-containing tripeptides unique to Gram-negative organisms, whereas the minimal molecule recognized by Nod2 is MDP, common to both Gram-negative and Gram-positive bacteria (27–29). To test whether PG changes resulting from a Δ*pgp2* mutation altered Nod receptor stimulation, we measured expression of a NF-κB *lgk* luciferase reporter transfected in human embryonic kidney HEK293T cells along with either the human Nod1 (Fig. 5*A*) or Nod2 (Fig. 5*B*) receptor and PG from either *C. jejuni* wild-type 81-176, Δ*pgp2* or Δ*pgp2*c. PG from Δ*pgp2* exhibited a statistically significant reduction in Nod1 stimulation in comparison with wild type at both concentrations tested. Specifically, stimulation with 0.2 μg of PG resulted in a 4.4-fold reduction for Δ*pgp2* in comparison with wild type, and stimulation with 2 μg of PG resulted in a 16.4-fold reduction. Nod1 activation by Δ*pgp2* PG even at the 0.2-μg concentration was also statistically significant in comparison with the non-stimulated negative control. PG from the complemented strain restored Nod1 activation to wild-type levels with the addition of 0.2 μg of PG and to nearly wild-type levels with 2 μg of PG. There was no statistically significant difference in Nod2 activation levels between the wild-type and Δ*pgp2* mutant PG (Fig. 5*B*).

Gentamicin protection assays were used to assess the ability of Δ*pgp2* to invade and survive intracellularly *in vitro* in the human epithelial cell lines INT407 (Fig. 5*C*) and Caco-2 (data not shown). The Δ*pgp2* mutant strain showed no defect in invasion or intracellular survival in comparison with wild type in either cell line. Because Δ*pgp2* has a motility defect in soft agar, we also assessed whether attachment and invasion to epithelial cells might be altered in a tissue culture medium of higher viscosity by the addition of CMC to the tissue culture media (11, 30). There was no significant difference in the ability of Δ*pgp2* in comparison with wild type to invade INT407 cells in the presence of 0.6, 1, or 2% CMC (data not shown).

Epithelial cells produce IL-8 in response to *C. jejuni* infection (31). The effect of loss of *pgp2* function on IL-8 secretion was examined by exposing the INT407 human epithelial cell line to *C. jejuni* wild type, Δ*pgp2*, and Δ*pgp2c* strains and measuring the levels of IL-8 in the supernatant after 8 and 24 h by ELISA (Fig. 5*D*). Data shown are representative of three separate experiments. There was no significant difference in the levels of IL-8 secreted from cells exposed to wild-type or Δ*pgp2* mutant strains.

To assess the role of *pgp2* in *in vivo* colonization, 1-day-old chicks were orally colonized with a dose of 10^4^ *C. jejuni* wild-type 81-176 and Δ*pgp2.* Levels of *C. jejuni* in the cecal contents were measured 7 days postcolonization. The Δ*pgp2* mutant exhibited a statistically significant (*p* = 0.0274) 2.1-log decrease in average levels of colonization compared with wild type (Fig. 5*E*).

## DISCUSSION

Elaborate mechanisms are involved in the preservation of bacterial morphology through growth and cell division, with morphology being maintained by the structure of the PG sacculus (8, 9). These mechanisms will differ depending on the shape of the organism. As predicted from its helical shape, *C. jejuni* must encode novel PG synthases or hydrolases involved in helical shape construction that are not found in rod-shaped bacteria. The PG dl-carboxypeptidase Pgp1 was the first unique helical shape determinant identified in *C. jejuni* (11). PG synthases and hydrolases are thought to be part of a multienzyme complex for PG assembly, with several interactions between PG enzymes and between PG enzymes and cytoskeletal elements having been reported (8–10, 32). Therefore, we hypothesized that identifying proteins interacting with Pgp1 might lead to the discovery of additional members of the *C. jejuni* morphogenesis program.

As with a *pgp1* mutant, deletion of *pgp2* resulted in a straight morphology and significant changes in its PG muropeptide profile. A direct physical interaction between Pgp1 and Pgp2 could not be shown experimentally through bacterial two-hybrid or pull-down assays.^6^ Enzymatic activity assays demonstrated that Pgp2 is an ld-carboxypeptidase cleaving monomeric and cross-linked PG tetrapeptides to tripeptides, despite lacking known carboxypeptidase domain(s). Pgp2 is predicted to have an N-terminal signal peptide and is probably periplasmic, acting on periplasmic PG sacculi. Although deletion of either *pgp1* or *pgp2* resulted in a loss of helical morphology in *C. jejuni*, only *pgp1* is conserved in bacteria with mainly curved or helical morphologies (11), whereas *pgp2* homologs are found in a wide variety of Gram-negative and -positive organisms. However, the precise distribution of Pgp2 is difficult to determine because similarity searches result in a list of organisms that encode for a YkuD or ld-transpeptidase catalytic domain, whereas Pgp2 was instead found to have ld-carboxypeptidase activity. ld-Carboxypeptidase activity has also been detected in many Gram-negative and positive species, although only a few enzymes with this specificity have been characterized (16). Bacterial ld-carboxypeptidases can either be cytoplasmic, cleaving tetrapeptides in the PG recycling pathway (*E. coli* LdcA (33, 34), *Pseudomonas aeruginosa* Pa5198 (35), and *Neisseria meningitidis* NMB1620 (36)) or, like Pgp2, have a signal peptide and be predicted to act in the periplasm on tetrapeptides in cross-linked PG sacculi (*Streptococcus pneumoniae* DacB (37) and *Lactococcus lactis* DacB (38)).

Consistent with Pgp2 enzymatic activity, the muropeptide profile of Δ*pgp2* shows a complete absence of tripeptide-containing muropeptides and an increase in tetrapeptides. The decrease in dipeptides is likewise expected because no tripeptides are present to serve as the substrate for the dl-carboxypeptidase Pgp1, which forms dipeptides from monomeric tripeptides. The muropeptide profile of the double mutant Δ*pgp1*Δ*pgp2* was identical to that of the single Δ*pgp2* mutant, further substantiating that Pgp2-mediated tripeptide formation is required for Pgp1 activity. Because deletion of *pgp2* eliminates the Pgp1 substrate, and *pgp1* is restricted to bacteria with curved or helical morphologies whereas *pgp2* is not, it is possible that Pgp1 alone is necessary for *C. jejuni* helical shape maintenance. There is still a residual amount of dipeptide (9–10%) present in the PG of both Δ*pgp2* and Δ*pgp1*Δ*pgp2*, suggesting that Pgp1 is not the only enzyme capable of forming dipeptide disaccharides and that *C. jejuni* probably encodes a hydrolase capable of cleaving pentapeptides or tetrapeptides to dipeptides that remains to be identified.

The shape of a bacterium has biological relevance (39, 40), and thus there is probably selective pressure for a bacterium like *C. jejuni* to retain its helical shape. It is therefore not surprising that *C. jejuni* pathogenic and survival attributes were altered when either *pgp1* or *pgp2* was deleted and helical morphology was lost. One consequence of *pgp2* deletion was a reduced motility in soft agar. The motility defect of Δ*pgp2* was reproducibly greater than that of Δ*pgp1*, possibly as a result of differences in the rod-shaped morphologies of the mutants affecting their swimming ability. Alternatively, despite what appears by TEM to be fully formed flagella on the cell surface of Δ*pgp1* and Δ*pgp2*, loss of PG hydrolases such as Pgp1 and, to a greater extent, Pgp2 may affect the proper assembly and/or function of the flagellar apparatus. Flagellar assembly may be less efficient than in the wild type because localized PG remodeling by PG hydrolases has been shown in other organisms to be required to create gaps in the PG layer permitting insertion of the multiprotein flagellar complex through the cell envelope (16, 41). An alternative explanation may be that flagellar function is compromised in the hydrolase mutants. In Gram-negative bacteria, PG interacts with the MotB protein of the flagellar MotA-MotB stator complex responsible for generating torque for flagellar rotation (42–45). In *H. pylori*, a mutant lacking the lytic transglycosylase MltD that cleaves the PG glycan backbone is non-motile, displaying a paralyzed phenotype with normally assembled flagella that are impaired in flagellar rotation and torque generation (46). Changes in PG structure resulting from Pgp1 and Pgp2 activities could alter interactions between MotB and PG residues, affecting the efficiency of flagellar rotation.

With *C. jejuni* motility being important for biofilm formation (47–49), the decreased motility of Δ*pgp2* may account for the modest biofilm defect. However, alterations in PG remodeling could also affect another *C. jejuni* factor required for biofilm development. Mutants in PG hydrolases or PG biosynthetic enzymes have also been shown to exhibit biofilm defects in several other organisms, such as *E. coli* (50, 51), *Burkholderia cepacia* (52), *Streptococcus gordonii* (53), and *L. lactis* (54); however, it also remains to be shown in these organisms what specific cell surface organelle or adhesin is affected. The Δ*pgp2* biofilm defect along with the hyporeactivity this mutant displayed on CFW further supports the link between CFW reactivity and key *C. jejuni* pathogenic properties, as described previously (11, 14, 26). CFW hyporeactivity of Δ*pgp2* was only apparent for the first 24 h following incubation, unlike that of Δ*pgp1*, which remained hyporeactive after 48 h. The Δ*pgp1* mutant was identified in a screen for hypofluorescent mutants after growth for 48 h on CFW, so it is not surprising that a mutant in *pgp2* was not isolated in that screen. The differences in CFW reactivity between Δ*pgp1* and Δ*pgp2* indicate that the mutants probably display different cell envelope properties during bacterial growth. One possible explanation may be that the mutants allow differential access to CFW binding sites on the PG during the aging process.

The changes in morphology, phenotypic properties, and PG muropeptide profile of Δ*pgp2* differ from wild type but also from Δ*pgp1* (11). These changes may also have unique effects on host interactions. Host epithelial cell innate immune responses to *C. jejuni* can be triggered by the activation of cytoplasmic Nod receptors recognizing bacterial muropeptides (55, 56). DAP-containing muropeptides serve as ligands for Nod1 receptors (57–59), with human Nod1 primarily recognizing tripeptides (60) and the motif recognized by human Nod2 being MDP (61, 62). The complete absence of tripeptide-containing muropeptides in Δ*pgp2* PG is reflected in the reduction of Nod1 activation in comparison with wild type. Despite a decrease in dipeptides, there was no change in Nod2 activation levels. This was also observed with the decreased levels of dipeptides in Δ*pgp1* PG (11). It is possible that Nod2 is not very sensitive to changes in levels of *C. jejuni* dipeptides. Our previous (11) and current work and that of Al-Sayeqh *et al.* (55) show that *C. jejuni* PG can activate Nod2, although that of Zilbauer *et al.* (56) did not. This may be the result of differences in the cell lines used or in reporter sensitivity.

A reduction in Nod1 activation by Δ*pgp2* muropeptides had no effect on the ability of Δ*pgp2* to survive intracellularly in *in vitro* epithelial cell infections or on the secretion of the proinflammatory mediator IL-8. IL-8 is released by intestinal epithelial cells in response to *C. jejuni* (63, 64) and can be triggered by Nod1 activation (56). Δ*pgp1* muropeptides hyperactivated Nod1, and IL-8 secretion did increase as a result of intestinal epithelial cell infection with the Δ*pgp1* strain, yet Δ*pgp2* muropeptides showed a drastic decrease in Nod1 activation with no effect on IL-8 levels secreted in response to the Δ*pgp2* strain in comparison with wild type. This could suggest that either only a certain base line of Nod1 activation is required for IL-8 secretion by epithelial cells, with further increases in IL-8 release resulting only when Nod1 activation rises beyond a certain threshold, or that other pathways in addition to Nod1 activation can result in IL-8 secretion (55, 64, 65). Therefore, the role of other bacterial factors, in addition to PG, in triggering IL-8 cannot be ruled out.

As with Δ*pgp1* and despite a more pronounced motility defect in soft agar, Δ*pgp2* was not impaired in host cell attachment, invasion, or intracellular survival *in vitro*, even in higher viscosity media. The helical morphology and polar flagella of *C. jejuni* are thought to be responsible for the high velocity darting motility observed by this organism in viscous media (7). Therefore, a loss of helical morphology and defect in soft agar motility would have been predicted to affect the ability of the organism to make contact with epithelial cells in higher viscosity media, yet Δ*pgp2* exhibited wild-type levels of epithelial cell attachment and invasion *in vitro*.

*C. jejuni* motility is also a key factor in *in vivo* colonization (22, 66–70), although our previous work showed that a decrease in motility alone was not sufficient to affect colonization in a Δ*carB* mutant (*carB* encodes carbamoylphosphate synthase), which, other than displaying a slight motility defect, exhibited helical shape and wild-type phenotypes for all other attributes tested (11). The *C. jejuni* helical shape and associated corkscrew motility have been hypothesized to be important in colonization, enabling *C. jejuni* to burrow through the mucosal layer. This is supported by the chick colonization defect of both Δ*pgp1* and Δ*pgp2* mutant strains that have lost their helical shape in addition to displaying reduced motility (11). *C. jejuni* colonization could be affected not only by a change in shape or in motility, but by the change in PG structure underlying the alteration in morphology. Despite both displaying straight morphology and having a similar effect on chick colonization, the Δ*pgp1* and Δ*pgp2* mutants have significantly different PG compositions, although both strains have a decrease in the level of dipeptides in comparison with wild type. Dipeptides are recognized by the Nod2 immune receptor, and although the chicken genome does not encode for a Nod2 receptor, it does possess an ortholog of the NLRP3/NALP3 Nod-like receptor that is similar to Nod2 and also binds MDP (71, 72). A more in depth understanding of chicken innate immunity and the role of dipeptides in signaling is required. Nonetheless, because Nod2 has been proposed to play a regulatory role in innate immune responses in mammalian systems (73), it is possible that reduced receptor signaling by decreased levels of dipeptides affects the chicken immune response to *C. jejuni* Δ*pgp1* and Δ*pgp2*, thereby altering colonization levels. It also cannot be ruled out that Δ*pgp1* and Δ*pgp2* mutations or changes to the PG structures in those mutants are affecting other, so far unidentified, bacterial cell envelope factors important in bacterial survival in the chick cecum or in host cell interactions mediating chicken immune responses.

Our ongoing characterization of PG synthesis or modification enzymes will provide additional insight into how helical shape is determined in *C. jejuni* and is necessary before a model can be proposed. We now know that at least Pgp1 and Pgp2 are required for helical twist in *C. jejuni*, although the addition of these enzymes to *E. coli* was not sufficient to transform *E. coli* from a rod to a helix shape,^5^ indicating that the presence of other proteins is required either for proper localization of Pgp1 and Pgp2 or for remodeling of the PG to act as a proper substrate. Other enzymes will probably affect the amount of helical curvature, with several candidates being the *C. jejuni* homologs of the *H. pylori* Csd1, CcmA, and Csd3/HdpA proteins (17, 18). The characterization of the Δ*pgp2* mutant as the second defined rod-shaped mutant after Δ*pgp1* serves to confirm the effects of the loss of helical morphology on the biology of *C. jejuni* described for Δ*pgp1*. However, it is difficult to attribute these defects solely to the loss of helical shape because changes in PG and the deletion of PG hydrolases can also have an effect on biological properties such as motility and biofilm formation. The PG muropeptides of Δ*pgp2* are distinct from those of Δ*pgp1* and differentially stimulate the epithelial cell Nod1 receptor. Because Nod activation is an important aspect of epithelial cell sensing to *C. jejuni* (56), continued studies with mutants with different muropeptide profiles will also lead to a better understanding of the effects of muropeptide structure on Nod sensing and their downstream effects on *C. jejuni* pathogenesis.

## Footnotes

5 E. Frirdich and E. C. Gaynor, unpublished observations.

6 E. Frirdich, J. Vermeulen, and E. C. Gaynor, unpublished observations.

## Footnotes

4 The abbreviations used are:

PG: peptidoglycan
CFW: calcofluor white
Nod: nucleotide-binding oligomerization domain
MurNAc: *N*-acetylmuramic acid
PBP: penicillin-binding protein
MH: Mueller-Hinton
Km: kanamycin
TEM: transmission electron microscopy
MDP: muramyldipeptide
DAP: diaminopimelic acid
CMC: carboxymethylcellulose.
